# Utility of high b-value (2000 sec/mm^2^) DWI with RESOLVE in differentiating papillary thyroid carcinomas and papillary thyroid microcarcinomas from benign thyroid nodules

**DOI:** 10.1371/journal.pone.0200270

**Published:** 2018-07-18

**Authors:** Qingjun Wang, Yong Guo, Jing Zhang, Lijing Shi, Haoyong Ning, Xiliang Zhang, Yuanyuan Lu

**Affiliations:** 1 Department of Radiology, Chinese Navy General Hospital of PLA, Beijing, China; 2 Department of Pathology, Chinese Navy General Hospital of PLA, Beijing, China; 3 Department of General Surgery, Chinese Navy General Hospital of PLA, Beijing, China; 4 Department of Ultrasound, Chinese Navy General Hospital of PLA, Beijing, China; University of South Alabama Mitchell Cancer Institute, UNITED STATES

## Abstract

**Purpose:**

The aim of the study was to evaluate the role of high b-value (2000 sec/mm^2^) diffusion-weighted imaging (DWI) by using Readout Segmentation of Long Variable Echo-trains (RESOLVE) in differentiating papillary thyroid carcinomas (PTCs) and papillary thyroid microcarcinomas (PTMCs) from benign thyroid nodules.

**Materials and methods:**

Consecutive patients with thyroid nodules scheduled for surgery underwent high b-value DWI with 3 b-values: 0, 800 and 2000 sec/mm^2^. Signal intensity ratios (SIRs) of thyroid nodules to adjacent normal thyroid tissue on DWI were measured as: SIR_b0_, SIR_b800_ and SIR_b2000_. Apparent diffusion coefficient (ADC) values based on the 3 different b-values were acquired as: ADC_b0-800_, ADC_b0-2000_, and ADC_b0-800-2000_. The 6 diagnostic indicators were evaluated by receiver operating characteristic (ROC) and diagnostic ability was compared between high b-value DWI and Ultrasound (US).

**Results:**

A total of 52 PTCs including 33 PTMCs (38 patients, 8 men and 30 women, aged 45.68 ± 11.93 years) and 62 benign thyroid nodules (46 patients, 7 men and 39 women, aged 48.73 ± 11.98 years) were enrolled into the final statistical analysis. ADC_b0-800-2000_ had the highest diagnostic ability in differentiating PTCs from benign thyroid nodules with area under curve (AUC) of 0.944, sensitivity of 96.15% and specificity of 85.48%, and PTMCs from benign thyroid nodules with AUC of 0.940, sensitivity of 93.94% and specificity of 85.48%. On the strength of lower false-positive rates than US (14.52% vs. 32.26% for PTCs and 14.52% vs. 32.26% for PTMCs), ADC_b0-800-2000_ had significantly better diagnostic ability in PTCs (*P* = 0.002) and PTMCs (*P* = 0.005).

**Conclusion:**

High b-value (2000 sec/mm^2^) DWI can contribute to differentiating PTCs and PTMCs from benign thyroid nodules and can be potentially used as an active surveillance imaging method for PTMCs.

## Introduction

Papillary thyroid carcinoma (PTC) is the most common malignant lesion of the thyroid gland, accounting for 90% of all thyroid malignant diseases [1]. When PTC is ≤ 10 mm in greatest diameter it is defined as papillary thyroid microcarcinoma (PTMC) according to World Health Organization (WHO) [2]. Ultrasound (US)-guided fine-needle aspiration biopsy (FNAB) is regarded as the most reliable diagnostic modality for thyroid nodules [3]. However, the recent American Thyroid Association (ATA) guidelines do not recommend diagnostic FNAB for PTMC and meanwhile underline additional studies are needed to search new reliable methods in determining specific risk factors to decide clinical management for PTMC [3].

By measuring the diffusion of water molecules, magnetic resonance (MR) diffusion-weighted imaging (DWI) can provide important information regarding tumor pathological features, such as cellular density and proliferative activity. Compared with benign tumors, malignant tumors often show more restricted diffusion of water molecules and decreased apparent diffusion coefficient (ADC), which contribute to the differentiation between benign and malignant tumors. Many studies have shown that DWI with conventional b-values (≤ 1000 sec/mm^2^) has the ability to differentiate malignant from benign thyroid nodules [4–8]. In DWI b-value is one of the most important parameters, because it is closely associated with image quality of DWI and diagnostic strength. By providing greater suppression of normal and benign tissues, high b-value (> 1000 sec/mm^2^) DWI can improve tumor visualization, diagnostic accuracy and grading [9–11]. However, high b-value DWI also faces technical challenge due to decreased signal-to-noise ratio (SNR) and increased anatomic distortion and artifacts, especially when used in superficial organs, such as the thyroid gland. Perhaps because of these problems the utility of high b-value DWI in differentiation between benign and malignant thyroid nodules have not been reported. DWI with sequence of multi-shot readout segmentation of long variable echo-trains (RESOLVE) can improve image quality of the neck and thyroid gland with less susceptibility artifacts, blurring from T2* signal decay and distortion [1, 12, 13]. This raises the possibility of diagnosing and differentiating thyroid nodules with high b-value (> 1000 sec/mm^2^) DWI to improve the diagnostic accuracy.

Previous studies have shown that high b-values in the range of 1500–2500 sec/mm^2^ are optimal for magnetic resonance imaging (MRI) diagnosis and differential diagnosis in prostate cancer [14, 15]. So far, no studies show diagnostic accuracy of high b-value (2000 sec/mm^2^) DWI in PTCs and PTMCs. Therefore, the aim of this study was to evaluate the role of high b-value (2000 sec/mm^2^) DWI using RESOLVE in discriminating PTCs and PTMCs from benign thyroid nodules.

## Materials and methods

### Population inclusion criteria

From June, 2015 to October, 2016, patients planned for US-guided FNAB or thyroid surgery due to thyroid nodules consecutively underwent the thyroid high b-value DWI. This study was approved by our institutional review board. Written consent was obtained from all patients before the study. Before MRI examination, all patients had been performed thyroid US (Philips IU 22, L12-5 ultrasonic probe, 7.5–10.0 MHz) and solid nodules or cystic and solid lesions were detected. There were two main reasons that these patients underwent the MRI examination before FNAB or thyroid surgery: (1) thyroid nodules had been classified by US as more than category 4a (suspiciously malignant) based on Thyroid Imaging Reporting and Data System (TI-RADS) [16] and surgeon wished to verify the US diagnosis by another way; or (2) although category 2 or 3 was considered by US on TI-RADS [16], thyroid nodules caused related symptoms (such as neck pressure, dysphagia, globus sensation, shortness of breath, dyspnea on exertion, and pain et al.), or had volume increase in spite of no related symptoms, or were more than 4 cm without related symptoms, and surgeon wished to clearly show the extent of the lesion before thyroid surgery. All patients underwent FNAB (by Y. Y. Lu who had 7 years’ experience in thyroid FNAB) or thyroid surgery (by X. L. Zhang who had 15 years’ experience in thyroid surgery) within 1 week after thyroid MRI examination. Nodules of papillary thyroid carcinoma and benign thyroid nodules proven by pathology would be enrolled into the study.

### Population exclusion criteria

**Fig 1:**
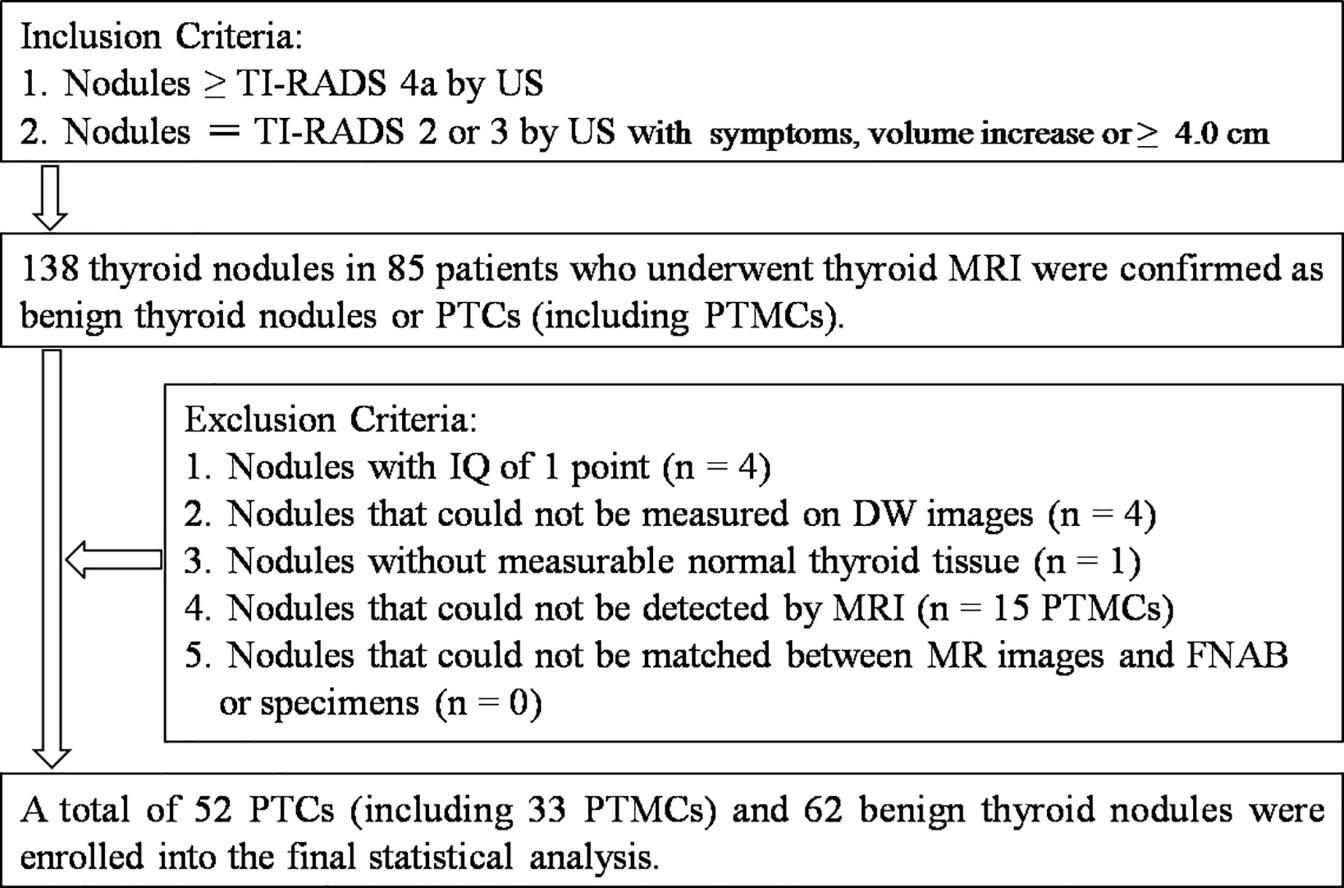
Flowchart of the study population. Abbreviations: TI-RADS, Thyroid Imaging Reporting and Data System; US, ultrasound; PTCs, papillary thyroid carcinomas; PTMCs, papillary thyroid microcarcinomas; IQ, image quality; FNAB, fine-needle aspiration biopsy.

Patients who have been performed thyroid FNAB or neck (including thyroid gland) radiotherapy, chemotherapy and surgery before MRI examination would be excluded from the study. Image quality (IQ) of each nodule on DWI with b-value of 2000 sec/mm^2^ was evaluated respectively according to 4-point scale depending on clarity of measured nodule: 4 = excellent (no problems were noticed and nodule was clearly shown), 3 = good (images suffered from only minor degradation and were suitable for the evaluation of nodule), 2 = moderate (images were not good but usable for evaluation of nodule), and 1 = poor (images precluded assessment of nodule and thyroid gland was barely shown). Nodules with IQ of 1 point would be excluded from the study. Nodules that couldn't be measured on DWI because of too tiny solid portions, massive hemorrhage and no normal thyroid tissue, and couldn't be detected by MRI or matched between MRI and FNAB or specimens would also be excluded. The study population is shown in Fig 1.

### Sample size determination

A recent meta-analysis about DWI with conventional b-values in differentiating malignant from benign thyroid nodules showed that the pooled weighted sensitivity and specificity were 90% and 95%, respectively [8]. Given that the diagnostic accuracy of high b-value DWI may be similar with or superior over that of DWI with conventional b-values, the sample size in this study was determined according to the following conventional diagnostic study formula: $N\;(PTC)=[{Z_{\alpha /2}}^{2}\times sensitivity\times (1-(sensitivity)]/\delta^{2}$ and $N\;(benign\;nodules)=[{Z_{\alpha /2}}^{2}\times specificity\times (1-specificity)]/\delta^{2}$, where the Z_α/2_ and δ were set to 1.96 and 0.1 respectively and both the sensitivity and specificity were assumed as 85%. Consequently, both the N (PTC) and N (benign nodules) were 49. Then at least 49 PTCs and 49 benign nodules should be included into the final analysis to support the statistical power of this study.

### MRI acquisition

All MRI examinations were performed with a 3.0T scanner (MAGNETOM Skyra, Siemens Healthcare, Erlangen, Germany) with a dedicated eight-channel bilateral surface coil for neck (Chenguang Medical Technologies CO., LTD, Shanghai, China) and a four-channel soft surface coil for thoracic entrance (Siemens Healthcare Sector, Erlangen, Germany). The protocol was acquired in the following order: coronal T2-weighted imaging (T2WI), axial T1-weighted imaging (T1WI) and T2WI, and axial high b-value DWI using 3 b-values. The high b-value DWI was performed using multi-shot echo-planar imaging (MS-EPI) sequence with RESOLVE and Generalized Autocalibrating Partially Parallel Acquisitions (GRAPPA) techniques. The averages of the high b-value DWI were 1 for b-value of 0 sec/mm^2^, 1 for b-value of 800 sec/mm^2^ and 4 for b-value of 2000 sec/mm^2^ and the second TE value (115 ms) belonged to a two dimensional (2D) navigator echo sequence which was used to eliminate signals with linear and non-linear phase errors, and robustly correct phase-induced motion artifacts. Three sets of DW images were automatically online acquired with the b-values of 0, 800 and 2000 sec/mm^2^. Meanwhile, by calculating apparent diffusion coefficient (ADC) in each pixel of each slice based on a mono-exponential model with the 3 sets of DW images [6], 3 sets of ADC maps were reconstructed without noise filtering: ADC_b0-800_, ADC_b0-2000_ and ADC_b0-800-2000_. The protocol parameters are shown in Table 1.

**Table 1:** Imaging protocol parameters of coronal T2WI, axial T1WI and T2WI, and axial high b-value DWI.

Parameters	Coronal T2WI	Axial T1WI	Axial T2WI	Axial high b-value DWI
Sequence	TSE	TSE	TSE	MS-EPI RESOLVE
Repetition time (ms)	3500	1490	3420	6200
Echo time (ms)	95	13	87	74/115
Fat suppression	Dixon	FS	FS	FS
Flip angle (°)	165	160	180	180
Section thickness (mm)	3	3	3	3
Section interval (mm)	0.3	0.3	0.3	0.3
Field of view (mm)	160	160	160	175
Matrix	224 × 320	256 × 320	256 × 320	174 × 174
Average	1	3	3	1, 1 and 4
Slices	24	24	24	24
Bandwidth (Hz/pixel)	300	250	260	685
Parallel acquisition factor	2	2	2	2
B-values (sec/mm^2^)	/	/	/	0, 800, and 2000
Readout segments	/	/	/	7
Acquisition time	2'13''	2' 34''	2' 39''	14' 11''

Abbreviations: T2WI, T2-weighted imaging; T1WI, T1-weighted imaging; DWI, diffusion-weighted imaging; TSE, turbo spin-echo; MS-EPI, multi-shot echo-planar imaging; RESOLVE, readout segmentation of long variable echo-trains; FS, frequency-selective.

### Image analysis

All images were transferred to a processing workstation (Syngo; Siemens). All assessments of IQ and measurements were performed in a consensus-oriented way by 2 radiologists (J. Zhang and L. J. Shi) who specialized in head and neck DWI and had more than 10 years’ experience in MRI applications. All images including the T1WI, T2WI and DWI were qualitatively reviewed first and solid nodules or solid component of cystic and solid lesions were identified. For cystic and solid lesions, only solid portion was evaluated and measured. The 2 radiologists were blinded to history, laboratory and pathological results of patients.

The 2 radiologists firstly determined the center of each solid nodule or the maximum solid component of each cystic and solid lesion, and located the center into one of 3 possible anatomic sites (isthmus, left or right lobe) and further precisely located each nodule according to the following methods: (1) longitudinally, coronal T2W image with the maximum transverse diameter (MTD) of the nodule would be selected and distances from the nodule’s center to thyroid upper and lower margins (D_MR_upper_ and D_MR_lower_) were recorded. Ratio (R) of D_MR_upper_/D_MR_lower_ (R_MR_UL_) was calculated. (2) Horizontally, axial DW image (b-value of 0 sec/mm^2^) of the nodule with MTD would be selected and distances from between the nodule’s center to thyroid nearest and farthest margins (D_nearest_ and D_farthest_) were recorded. R of D_nearest_/D_farthest_ (R_MR_NF_) was also calculated.

The slice with MTD of each nodule on DWI with b-value of 0 sec/mm^2^ was selected and a region of interest (ROI) was drawn to encompass the whole outline of a solid nodule or the maximum solid component of a cystic and solid lesion on the slice. The ROI was carefully drawn to avoid hemorrhage, necrosis, calcium, cystic changes and vascular structures and copied to the corresponding DW images with b-values of 800 and 2000 sec/mm^2^ and 3 sets of ADC maps (ADC_b0-800_, ADC_b0-2000_ and ADC_b0-800-2000_). Besides the 3 ADC values, the values of nodule's signal intensity (SI_nodule_) with b-values of 0, 800 and 2000 sec/mm^2^ were acquired: SI_nodule_b0_, SI_nodule_b800_ and SI_nodule_b2000_. Another ROI with an area of about 1.0 cm^2^ was place on normal thyroid tissue adjacent to the nodule in the same slice to acquire the SI of normal thyroid tissue with b-values of 0, 800 and 2000 sec/mm^2^: SI_NTT_b0_, SI_NTT_b800_ and SI_NTT_b2000_. If normal thyroid tissue could not be found in area adjacent to the nodule, the ROI of SI_NTT_ would be placed in the contralateral normal thyroid tissue in the same slice. For the thyroid gland with hashimoto's thyroiditis, ROIs of SI_NTT_ would be placed in area where no nodules could be distinguishable with naked eyes. R of SI_nodule_/SI_NTT_ was respectively calculated on DW images with b-values of 0, 800 and 2000 sec/mm^2^: SIR_b0_ = SI_nodule_b0_/SI_NTT_b0_, SIR_b800_ = SI_nodule_b800_/SI_NTT_b800_ and SIR_b2000_ = SI_nodule_b2000_/SI_NTT_b2000_. MTD of each nodule on DW images with b-value of 0 sec/mm^2^ was measured as DWI-MTD.

### Pathology of nodules on MR images

#### Locating nodules in FNAB

The US doctor (Y. Y. Lu) located each nodule examined by FNAB according to the same methods used by the radiologists who located nodules on MR images longitudinally and horizontally: longitudinal distances from the nodule’s center to thyroid upper and lower margins on US (D_US_upper_ and D_US_lower_) and horizontal distances from the nodule’s center to thyroid nearest and farthest margins on US (D_US_nearest_ and D_US_farthest_). Both R of D_US_upper_/D_US_lower_ (R_US_UL_) and R of D_US_nearest_/D_US_farthest_ (R_US_NF_) were calculated. If a nodule was confirmed as a benigncy, it would be followed up and its' location in FNAB (R_US_UL_ and R_US_NF_) would be recorded for matching with that on MR images. If a nodule was confirmed as a malignancy, it would be planned for total thyroidectomy.

#### Locating nodules in specimens

If a nodule, which had been diagnosed as ≥ TI-RADS 4a by US, was confirmed as a malignancy by FNAB before surgery, total thyroidectomy would be given directly and the specimen of total thyroidectomy would be acquired. If a nodule, which had been diagnosed as ≥ TI-RADS 4a by US, was not performed FNAB before surgery, lobectomy would be given first for intraoperative frozen section to determine the nodule's nature. If the nodule was confirmed as a benigncy by intraoperative frozen section, total thyroidectomy would not be applied and the specimen of lobectomy would be acquired. On the contrary, if the nodule was confirmed as a malignancy, total thyroidectomy would be further given and the specimen of total thyroidectomy would be acquired. Nodules, which had been diagnosed as TI-RADS 2 or 3, would first undergo lobectomy or subtotal thyroidectomy for intraoperative frozen section to determine the nodule's nature. If the nodule was proven as a benigncy by intraoperative frozen section, total thyroidectomy would not be applied and the specimen of lobectomy or subtotal thyroidectomy would be acquired. On the contrary, if the nodule was proven as a malignancy, total thyroidectomy would be further given and the specimen of total thyroidectomy would be acquired.

An experienced pathologist (H. Y. Ning) cut thyroid specimens from lobectomy, subtotal thyroidectomy or total thyroidectomy into slices from upper to lower pole layer-by-layer every other 3.0–3.5 mm similar with the thickness and interval of axial MR images. The pathologist carefully sought any nodule in each slice and isolated the slice, in which the pathological MTD of solid portion of nodules were measured before pathological paraffin. The pathologist recorded longitudinal and horizontal location information of each nodule's center according to the same methods used by the radiologists who located nodules on MR images: longitudinal distances from the nodule’s center to thyroid upper and lower margins on specimen (D_specimen_upper_ and D_specimen_lower_) and horizontal distances from the nodule’s center to thyroid nearest and farthest margins on specimen (D_specimen_nearest_ and D_specimen_farthest_). Both R of D_specimen_upper_/D_specimen_lower_ (R_specimen_UL_) and R of D_specimen_nearest_/D_specimen_farthest_ (R_specimen_NF_) were calculated.

#### Matching nodules between MR images and FNAB or specimens

For benign nodules proven by FNAB and didn't undergo surgery, the 2 radiologists and the US doctor together matched these nodules between MR images and specimens by comparing the deviation between location data: (1) percentage (%) of the difference value (D) between R_US_UL_ to R_MR_UL_ (D_RUS_MR_UL_) against R_US_UL_ (D_RUS_MR_UL_/R_US_UL_%) and R_MR_UL_ (D_RUS_MR_UL_/R_MR_UL_%), and (2) percentage (%) of the difference value (D) between R_US_NF_ to R_MR_NF_ (D_RUS_MR_NF_) against R_US_NF_ (D_RUS_MR_NF_/R_US_UL_%) and R_MR_NF_ (D_RUS_MR_NF_/R_MR_NF_%). Given potential measuring error, if all the absolute values of D_RUS_MR_UL_/R_US_UL_%, D_RUS_MR_UL_/R_MR_UL_%, D_RUS_MR_NF_/R_US_NF_% and D_RUS_MR_NF_/R_MR_NF_% weren't more than 10%, a successful match would be recognized.

For these nodules by lobectomy, subtotal thyroidectomy or total thyroidectomy, the 2 radiologists, surgeon and pathologist together matched these nodules between MR images and specimens by comparing the deviation between location data: (1) percentage (%) of the difference value (D) between R_specimen_UL_ to R_MR_UL_ (D_Rspecimen_MR_UL_) against R_specimen_UL_ (D_Rspecimen_MR_UL_/R_specimen_UL_%) and R_MR_UL_ (D_Rspecimen_MR_UL_/R_MR_UL_%), and (2) percentage (%) of the difference value (D) between R_specimen_NF_ to R_MR_NF_ (D_Rspecimen_MR_NF_) against R_specimen_NF_ (D_Rspecimen_MR_UL_/R_specimen_NF_%) and R_MR_NF_ (D_Rspecimen_MR_UL_/R_MR_NF_%). In consideration of the 10%-15% shrinkage of in-vitro thyroid gland, if all the absolute values of D_Rspecimen_MR_UL_/R_specimen_UL_%, D_Rspecimen_MR_UL_/R_MR_UL_%, D_Rspecimen_MR_UL_/R_specimen_NF_% and D_Rspecimen_MR_UL_/R_MR_NF_% weren't more than 20%, a successful match would also be recognized for a nodule.

### Statistical analysis

One-Sample Kolmogorov-Smirnov Test was used firstly for analysis of the normality of continuous variables. Quantitative variables with normal distribution were expressed as mean ± standard deviation (SD). Quantitative variables without normal distribution were expressed as median (range). Qualitative variables were expressed as percentages. All lesions of papillary thyroid carcinoma were divided into 2 subgroups according to the pathological MTD: PTCs (pathological MTD > 10 mm) and PTMCs (pathological MTD ≤ 10 mm). The Student unpaired *t* test (or Welch test for unequal variances) or χ^2^ test was applied to compare the difference of continuous variables or qualitative variables between the benign and malignant subgroups. In Boxplot, the far outside value was defined as the third quartile (Q3) + 3 interquartile range (IQR) or the first quartile (Q1) - 3 (IQR). The receiver operating characteristic curve (ROC) was used to obtain the diagnostic threshold, area under curve (AUC), sensitivity, specificity of these diagnostic indicators. The diagnostic ability among diagnostic indicators in the high b-value DWI and between the high b-value DWI and US was further compared by ROC comparison analysis. Six months after the first measurements of SIR and ADC, all these matched nodules were reviewed again by another two readers (Q. J. Wang and Y. Guo), who intended to calculate interobserver variability by means of intraclass correlation coefficient (ICC). All statistical analysis were performed with MedCalc Software, version 11.4.2.0 (MedCal Software, Mariakerke, Belgium) and the level of statistical significance was determined by *P* < 0.05.

## Results

### Clinical-pathologic findings and matching results

A total of 138 thyroid nodules in 85 patients who underwent thyroid MRI were confirmed as benign thyroid nodules or PTCs by pathology after FANB or surgery. Ten patients had both benign nodules (n = 16) and PTCs (n = 20). There were 68 benign thyroid nodules in 51 patients (10 men and 41 women) and 70 PTCs (including 49 PTMCs) in 44 patients (13 men and 31 women). Seventeen PTCs were complicated with hashimoto's thyroiditis and 19 patients with PTCs had lymphatic metastasis in at least one side central region. Four simple nodular goiters (in 4 patients) were confirmed by FANB and weren't performed thyroid surgery. Nine PTCs (in 8 patients) were confirmed by FANB and then were performed thyroid surgery. The other nodules were confirmed by postoperative pathology. All the 68 benign thyroid nodules proven by pathology could be matched on MRI images. For the 4 simple nodular goiters confirmed by FANB, the minimum and maximum were 6.23% and 12.16% in D_RUS_MR_UL_/R_US_UL_%, 12.01% and 6.64% in D_RUS_MR_UL_/R_MR_UL_%, 2.03% and 7.13% in D_RUS_MR_NF_/R_US_NF_%, 2.07% and 6.66% in D_RUS_MR_NF_/R_MR_NF_%. For the other 64 benign thyroid nodules underwent lobectomy or subtotal thyroidectomy, the mean ± SD were 7.79% ± 4.34% in D_Rspecimen_MR_UL_/R_specimen_UL_%, 7.94% ± 4.54% in D_Rspecimen_MR_UL_/R_MR_UL_%, 11.71% ± 5.22% in D_Rspecimen_MR_UL_/R_specimen_NF_%, and 10.48% ± 4.24% in D_Rspecimen_MR_UL_/R_MR_NF_%. Among the 70 PTCs (including 49 PTMCs), a total of 15 PTMCs could be detected neither by MRI nor by US with the pathological MTD: 0.5 mm (1 lesion), 0.7 mm (2 lesions), 0.8 mm (1 lesion), 1.0 mm (5 lesions), 1.5mm (1 lesion), and 2.0 mm (5 lesions). The other 55 PTCs (including 34 PTMCs) proven by histology could be matched with MR images. The mean ± SD were 8.29% ± 5.18% in D_Rspecimen_MR_UL_/R_specimen_UL_%, 8.20% ± 5.15% in D_Rspecimen_MR_UL_/R_MR_UL_%, 9.83% ± 5.13% in D_Rspecimen_MR_UL_/R_specimen_NF_%, and 9.06% ± 4.44% in D_Rspecimen_MR_UL_/R_MR_NF_%.

### Synopsis of nodules on high b-value DWI

The median (range) of IQ for the benign nodules was 4 (1–4). A total of 6 benign nodules in 6 patients were excluded from the study: 3 nodules for too tiny solid portions to be measured, 1 for massive hemorrhage, 1 for no normal thyroid tissue and 1 for the IQ with 1 point. Therefore, 62 benign thyroid nodules in 46 patients (7 men and 39 women, aged 48.73 ± 11.98 years) were enrolled into the statistical analysis: 43 simple nodular goiters, 10 nodular goiters complicated by adenomas, 4 nodular goiters complicated by hashimoto's thyroiditis, 2 nodular goiters complicated by hashimoto's thyroiditis and adenomas, and 3 nodular hashimoto's thyroiditis. There were a total of 9 (9/62, 14.52%) benign thyroid nodules related with hashimoto's thyroiditis (complicated by hashimoto's thyroiditis or nodular hashimoto's thyroiditis). The mean pathological MTD of benign nodules was 23.79 ± 18.32 mm and the corresponding DWI-MTD was 23.55 ± 18.11 mm. There was no statistical difference in MTD of these matched benign nodules between the histology and DWI (*t* = -0.836, *P* = 0.407).

The median (range) of IQ for all these matched PTCs was 4 (1–4). Three PTCs in 3 patients were excluded from the study: 2 PTCs with 1 point IQ due to neck obesity in 2 patients (pathological MTD of 9 and 11 mm, respectively) and 1 PTC with 1 point IQ due to motion artifacts in 1 patient (pathological MTD of 17 mm). Therefore, a total of 52 PTCs (including 33 PTMCs) in 38 patients (8 men and 30 women, aged 45.68 ± 11.93 years) were enrolled into the statistical analysis. The mean pathological MTD of all the 52 PTCs was 10.65 ± 6.77 mm and the corresponding DWI-MTD was 11.16 ± 7.29 mm. There was no statistical difference in MTD of these matched PTCs between the histology and DWI (*t* = 1.038, *P* = 0.304). The mean pathological MTD of the 33 PTMCs was 6.79 ± 2.55 mm and the corresponding DWI-MTD was 7.58 ± 3.22 mm. No statistical difference in MTD of these matched PTMCs between the histology and DWI was found (*t* = -1.112, *P* = 0.270).

### Comparison of diagnostic indicators in high b-value DWI between nodules of papillary thyroid carcinoma and benign thyroid nodules

**Fig 2:**
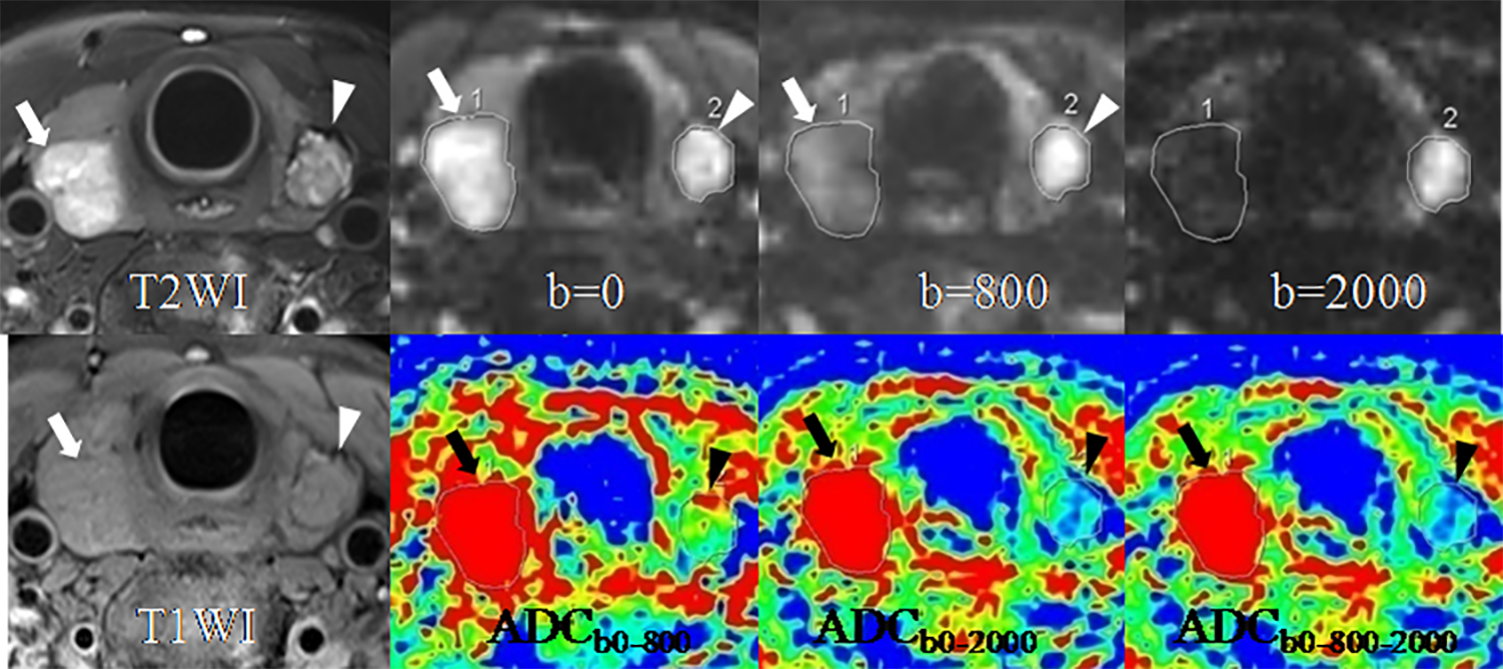
A goiter in the right lobe (arrows) and a papillary thyroid carcinoma (arrow-heads) in the left lobe in a 42-year-old woman. SIR of the goiter decreases while SIR of the papillary carcinoma increases with the b-value from 0 to 800 and 2000 sec/mm^2^. ADC of the goiter is obviously higher than that of the papillary carcinoma, especially on the ADC maps of ADC_b0-2000_ and ADC_b0-800-2000_. Abbreviations: SIR, signal intensity ratio; ADC, apparent diffusion coefficient.

**Fig 3:**
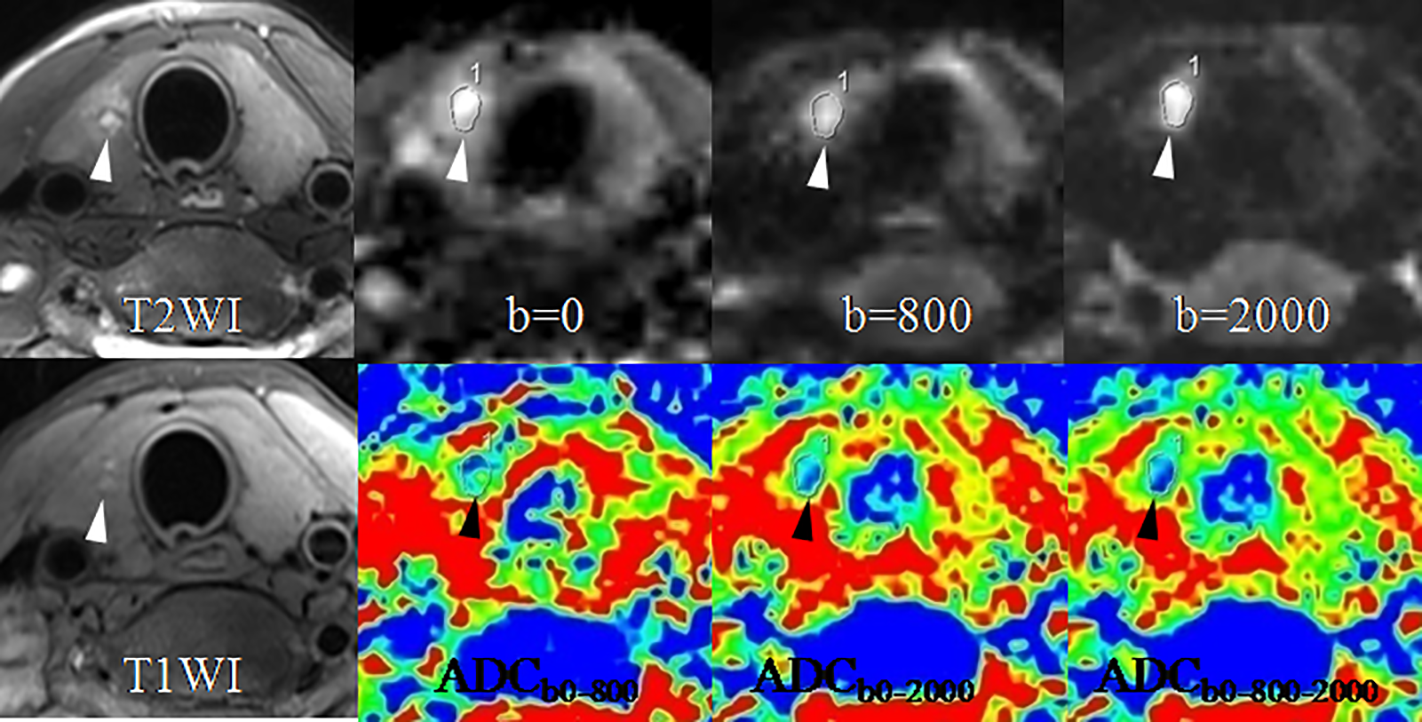
A papillary thyroid microcarcinoma (arrow-heads) in the right lobe in a 26-year-old woman. SIR of the lesion increases with the b-value from 0 to 800 and 2000 sec/mm^2^. ADC of the lesion is markedly lower than that of the normal thyroid tissue, especially on the ADC maps of ADC_b0-2000_ and ADC_b0-800-2000_. Abbreviations: ADC, apparent diffusion coefficient.

**Fig 4:**
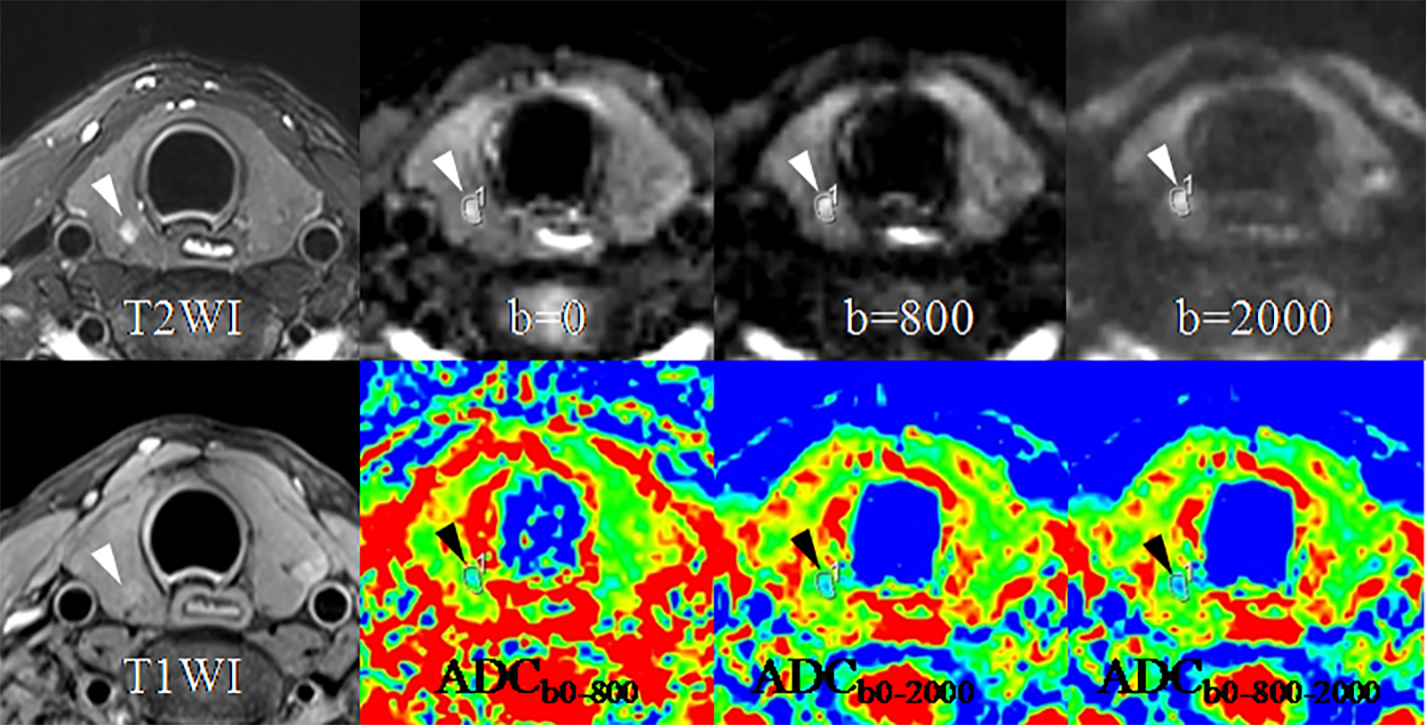
A papillary thyroid microcarcinoma (arrow-heads) in the right lobe in a 35-year-old woman. SIR of the lesion rises with the b-value from 0 to 800 and 2000 sec/mm^2^. ADC of the lesion drops on the ADC maps of ADC_b0-2000_ and ADC_b0-800-2000_ compared with on that of ADC_b0-800_. Abbreviations: ADC, apparent diffusion coefficient.

**Fig 5:**
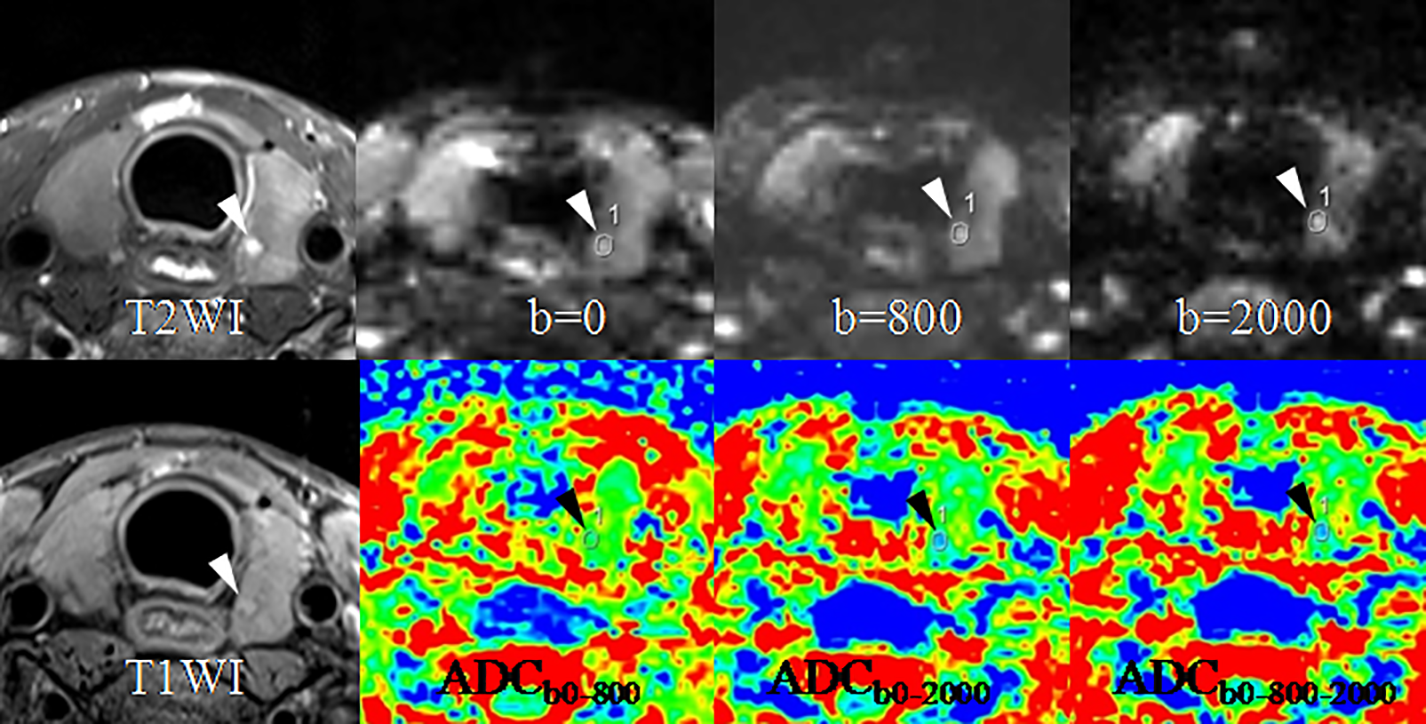
A papillary thyroid microcarcinoma (arrow-heads) in the left lobe in a 25-year-old woman. SIR ascent of the lesion can be seen with the b-value from 0 to 800 and 2000 sec/mm^2^. ADC descent of the lesion on the ADC maps of ADC_b0-2000_ and ADC_b0-800-2000_ also can be noted compared with on that of ADC_b0-800_. Abbreviations: ADC, apparent diffusion coefficient.

Significant differences were observed in all the 6 diagnostic indicators between the 2 groups (all *P* < 0.05, Table 2). PTCs showed lower SIR_b0_ but higher SIR_b800_ and SIR_b2000_, and lower ADCs (including ADC_b0-800_, ADC_b0-2000_ and ADC_b0-800-2000_) than benign thyroid nodules (Table 2). PTMCs were significantly different in SNR_0_, SNR_2000_, ADC_b0-800_, ADC_b0-2000_, and ADC_b0-800-2000_ compared with benign thyroid nodules (all *P* < 0.001, Table 2). PTMCs also showed lower SNR_b0_ but higher SNR_b2000_, and lower ADCs (including ADC_b0-800_, ADC_b0-2000_ and ADC_b0-800-2000_) than benign thyroid nodules (Table 2). Figs 2–5 show the differences between PTCs (including PTMCs) and benign nodule in SNR and ADC.

**Table 2:** Comparison of diagnostic indicators between nodules of papillary thyroid carcinoma and benign thyroid nodules.

Diagnostic indicators	Benign thyroid nodules (n = 62)	PTCs(n = 52)	*P*	PTMCs (n = 33)	*P*
SIR_b0_	2.01 ± 1.03	1.32 ± 0.46	< 0.001*	1.17 ± 0.30	< 0.001*
SIR_b800_	1.53 ± 0.95	1.93 ± 0.98	0.031	1.62 ± 0.61	0.586*
SIR_b2000_	1.23 ± 1.03	2.29 ± 1.21	< 0.001	1.92 ± 0.74	< 0.001*
ADC_b0-800_, mm^2^/sec	1.99 ± 0.45	1.30 ± 0.30	< 0.001*	1.31 ± 0.30	< 0.001*
ADC_b0-2000_, mm^2^/sec	1.43 ± 0.27	0.92 ± 0.16	< 0.001*	0.94 ± 0.17	< 0.001*
ADC_b0-800-2000_, mm^2^/sec	1.40 ± 0.27	0.89 ± 0.16	< 0.001*	0.90 ± 0.16	< 0.001*

* Welch-test assuming unequal variances. Abbreviations: PTCs, papillary thyroid carcinomas; PTMCs, papillary thyroid microcarcinomas; SIR, signal intensity ratio; ADC, apparent diffusion coefficient.

**Fig 6:**
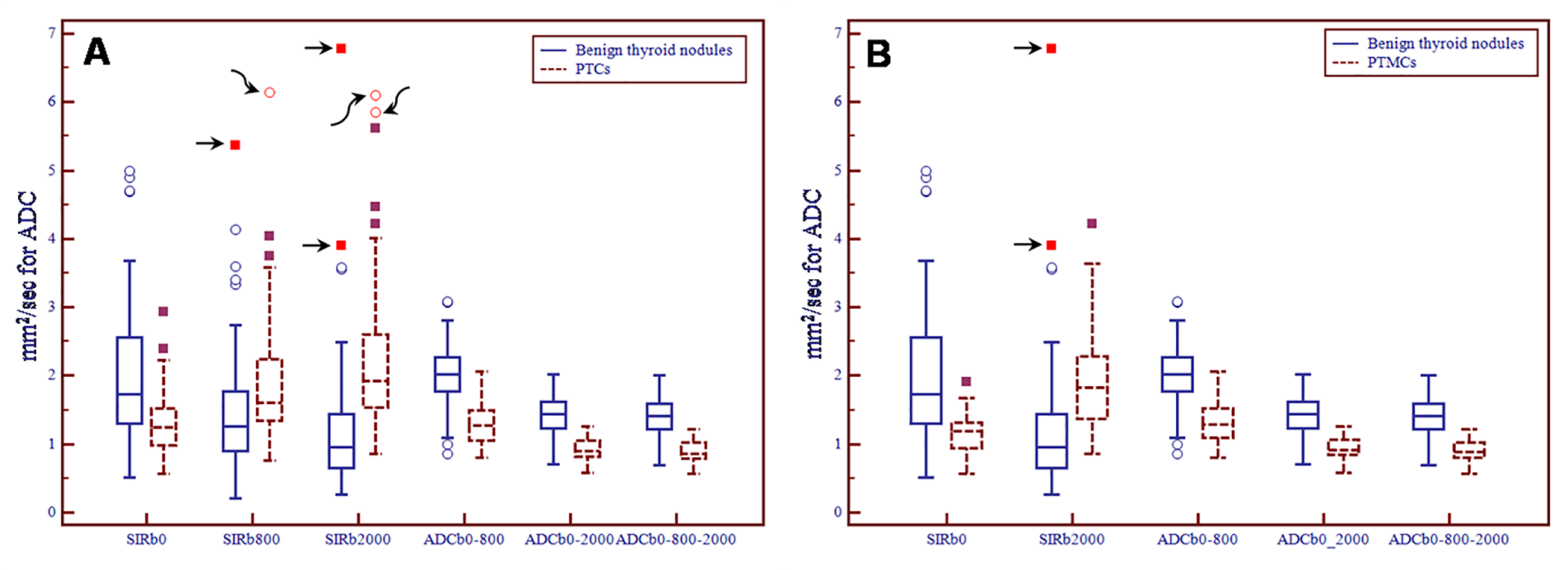
**Boxplot diagrams of significant comparison in diagnostic indicators between thyroid benign nodules and nodules of papillary thyroid microcarcinoma (A, PTCs and B, PTMCs).** There are 5 far outside values in the benign thyroid nodules (black arrows) and 3 in the PTCs (curved black arrows) in the 2 Boxplot diagrams. All the 5 far outside values in the benign thyroid nodules are simple nodular hashimoto's thyroiditis. Abbreviations: SIR, signal intensity ratio; ADC, apparent diffusion coefficient; PTCs, papillary thyroid carcinomas; PTMCs, papillary thyroid microcarcinomas.

According to Table 2, Boxplot diagrams were also made to analyze the far outside values that maybe had a potentially great influence on the comparison of diagnostic indicators between PTCs or PTMCs and benign thyroid nodules (Fig 6). For comparison between PTCs and benign thyroid nodules (Fig 6A), in SIR_b800_ a far outside value was a simple nodular hashimoto's thyroiditis in benign thyroid nodules, which narrowed the contrast and another far outside value is a PTC in malignant nodules, which expanded the contrast. In SIR_b2000_ 2 far outside values were 2 simple nodular hashimoto's thyroiditis in benign thyroid nodules, which narrowed the contrast and another 2 far outside values were 2 PTCs in malignant nodules, which expanded the contrast. For comparison between PTMCs and benign thyroid nodules (Fig 6B), in SIR_b2000_ 2 far outside values were 2 simple nodular hashimoto's thyroiditis in benign thyroid nodules, which narrowed the contrast. Therefore, in the Boxplot diagrams there were 8 far outside values and 5 of them were simple nodular hashimoto's thyroiditis from benign thyroid nodules, which narrowed the contrast in SIR_b800_ and SIR_b2000_.

### ROC analysis of diagnostic indicators in differentiating nodules of papillary thyroid carcinoma from benign thyroid nodules

**Fig 7:**
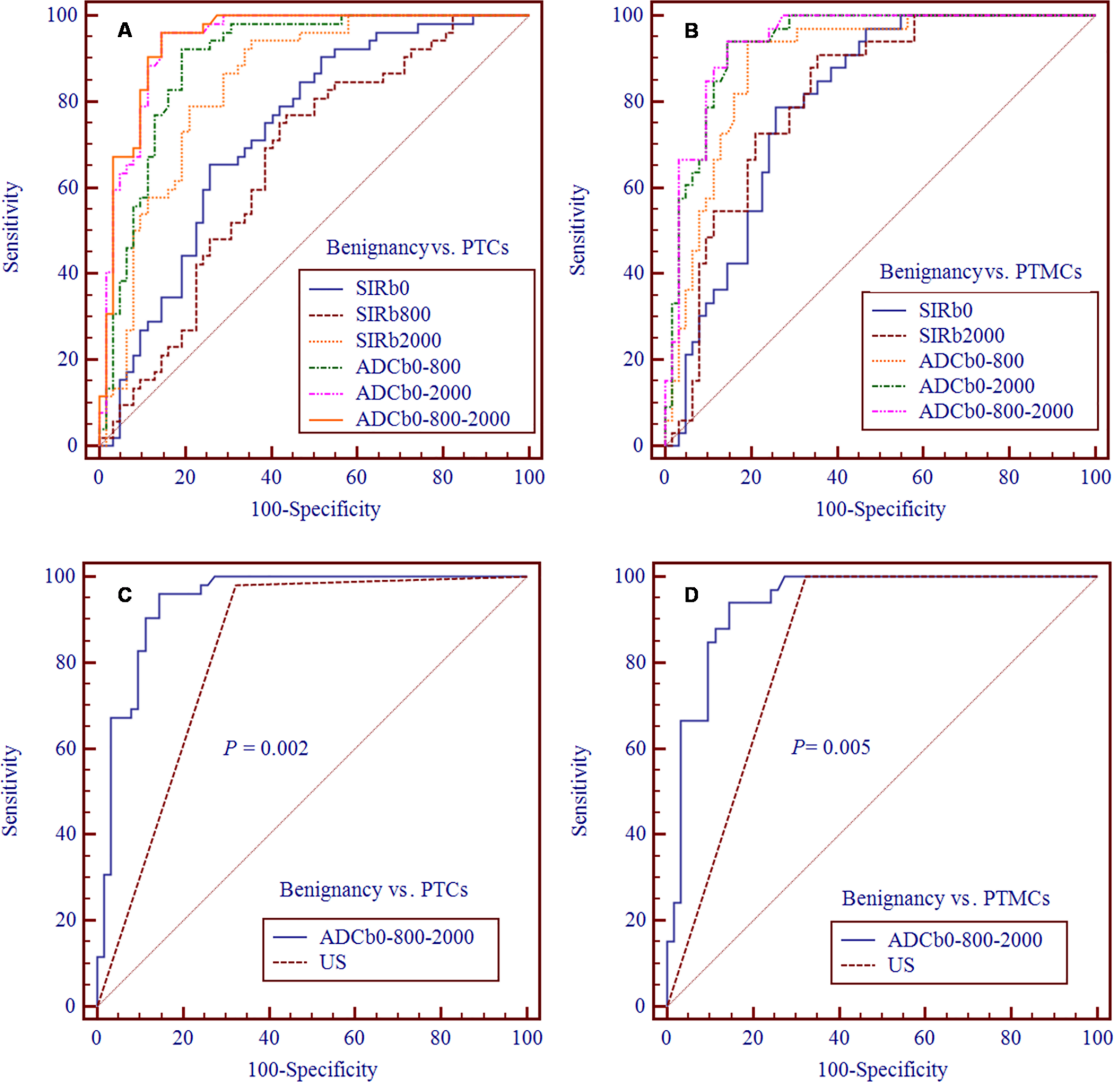
**Comparison of ROC curves between the diagnostic indicators in differentiating thyroid benign nodules from nodules of papillary thyroid microcarcinoma (A, PTCs and B, PTMCs) and comparison of ROC curves between ADC**_**b0-800-2000**_ **and US (C, PTCs and D, PTMCs).** Abbreviations: SIR, signal intensity ratio; ADC, apparent diffusion coefficient; PTCs, papillary thyroid carcinomas; PTMCs, papillary thyroid microcarcinomas; US, ultrasound.

For discriminating PTCs from benign thyroid nodules, among the 3 diagnostic indicators of SIR (SIR_b0_, SIR_b800_ and SIR_b2000_), the sensitivity of SNR_b2000_ was highest (94.23%). Although AUC of SNR_b2000_ was significantly higher than that of SNR_b8000_ (AUC of SIR_b800_ vs. AUC of SIR_b2000_: 0.660 vs. 0.842, *P <* 0.001, Table 3 and Table 4), it showed no significant difference compared with SNR_b0_ (AUC of SIR_b0_ vs. AUC of SIR_b2000_: 0.730 vs. 0.842, *P* = 0.112, Table 3 and Table 4). Except for ADC_b0-2000_ (AUC: 0.940, *P* = 0.364), ADC_b0-800-2000_ had the significantly higher AUC (0.944) than SIR_b0_ (AUC: 0.730, *P* < 0.001), SIR_b800_ (AUC: 0.660, *P <* 0.001), SIR_b2000_ (AUC: 0.842, *P* = 0.012) and ADC_b0-800_ (AUC: 0.896, *P* = 0.026) (Table 3 and Table 4). If ADC_b0-800-2000_ ≤ 1.16 mm^2^/sec was adopted as the cutoff value, the sensitivity and specificity were 96.15% and 85.48%, respectively (Table 3). Fig 7A shows the comparison of ROC curves between SIR_b0_, SIR_b800_, SIR_b2000_, ADC_b0-800_, ADC_b0-2000_, and ADC_b0-800-2000_ in differentiating PTCs from benign thyroid nodules.

**Table 3:** ROC analysis of diagnostic indicators in differentiating nodules of papillary thyroid carcinoma from benign thyroid nodules.

Subgroups	Diagnostic indicators	Cutoff value	AUC	95% CI	Sensitivity (%)	95% CI	Specificity (%)	95% CI
PTCs (n = 52)	SIR_b0_	≤ 1.32	0.730	0.639–0.809	65.38	50.9–78.0	74.19	61.5–84.5
	SIR_b800_	> 1.29	0.660	0.565–0.746	76.92	63.2–87.5	56.45	43.3–69.0
	SIR_b2000_	> 1.17	0.842	0.761–0.903	94.23	84.1–98.8	64.52	51.3–76.3
	ADC_b0-800_, mm^2^/sec	≤ 1.69	0.896	0.825–0.946	92.31	81.5–97.9	80.65	68.6–89.6
	ADC_b0-2000_, mm^2^/sec	≤ 1.18	0.940	0.880–0.976	96.15	86.8–99.5	85.48	74.2–93.1
	ADC_b0-800-2000_, mm^2^/sec	≤ 1.16	0.944	0.884–0.978	96.15	86.8–99.5	85.48	74.2–93.1
PTMCs (n = 33)	SIR_b0_	≤ 1.32	0.797	0.702 - 0.873	78.79	61.1–91.0	74.19	61.5–84.5
	SIR_b2000_	>1.17	0.814	0.721 - 0.886	90.91	75.7–98.1	64.52	51.3–76.3
	ADC_b0-800_, mm^2^/sec	≤ 1.67	0.892	0.811 - 0.946	93.94	79.8–99.3	80.65	68.6–89.6
	ADC_b0-2000_, mm^2^/sec	≤ 1.18	0.935	0.865 - 0.975	93.94	79.8–99.3	85.48	74.2–93.1
	ADC_b0-800-2000_, mm^2^/sec	≤ 1.16	0.940	0.871 - 0.978	93.94	79.8–99.3	85.48	74.2–93.1

Abbreviations: PTCs, papillary thyroid carcinomas; PTMCs, papillary thyroid microcarcinomas; SIR, signal intensity ratio; ADC, apparent diffusion coefficient; AUC, area under curve; CI, confidence interval.

**Table 4:** Comparison of the diagnostic ability between these diagnostic indicators by pair-wise ROC analysis.

Subgroups	Diagnostic indicators *	SIR_b0_	SIR_b800_	SIR_b2000_	ADC_b0-800_, mm^2^/sec	ADC_b0-2000_, mm^2^/sec	ADC_b0-800-2000_, mm^2^/sec
PTCs (n = 52)	SIR_b0_	-	0.419	0.112	0.002	< 0.001	< 0.001
	SIR_b800_	0.419	-	< 0.001	< 0.001	< 0.001	< 0.001
	SIR_b2000_	0.112	< 0.001	-	0.181	0.016	0.012
	ADC_b0-800_, mm^2^/sec	0.002	< 0.001	0.181	-	0.025	0.026
	ADC_b0-2000_, mm^2^/sec	< 0.001	< 0.001	0.016	0.025	-	0.364
	ADC_b0-800-2000_, mm^2^/sec	< 0.001	< 0.001	0.012	0.026	0.364	-
PTMCs (n = 33)	SIR_b0_	-		0.817	0.064	0.002	0.001
	SIR_b2000_	0.817		-	0.103	0.009	0.006
	ADC_b0-800_, mm^2^/sec	0.064		0.103	-	0.047	0.041
	ADC_b0-2000_, mm^2^/sec	0.002		0.009	0.047	-	0.306
	ADC_b0-800-2000_, mm^2^/sec	0.001		0.006	0.041	0.306	-

* The results of the pairwise ROC comparison were *P* values. Abbreviations: PTCs, papillary thyroid carcinomas; PTMCs, papillary thyroid microcarcinomas; SIR, signal intensity ratio; ADC, apparent diffusion coefficient.

For discriminating PTMCs from benign thyroid nodules, SIR_b2000_ was not significantly different from SNR_b0_ in diagnostic ability (AUC of SIR_b0_ vs. AUC of SIR_b2000_: 0.797 vs. 0.814, *P* = 0.817, Table 3 and Table 4), whereas, a reverse contrast of sensitivity and specificity between SIR_b0_ and SIR_b2000_ was found. The sensitivity of SIR_b2000_ was higher than that of SIR_b0_ and the specificity of SIR_b2000_ was lower than that of SIR_b0_ (Table 3). Except for ADC_b0-2000_ (AUC: 0.935, *P* = 0.306), ADC_b0-800-2000_ had the significantly higher AUC (0.940) than SIR_b0_ (AUC: 0.797, *P <* 0.001), SIR_b2000_ (AUC: 0.814, *P* = 0.006) and ADC_b0-800_ (AUC: 0.892, *P* = 0.041) (Table 3 and Table 4). If ADC_b0-800-2000_ ≤ 1.16 mm^2^/sec was adopted as the cutoff value, the sensitivity and specificity were 93.94% and 85.48%, respectively (Table 3). Fig 7B shows the comparison of ROC curves between SIR_b0_, SIR_b2000_, ADC_b0-800_, ADC_b0-2000_, and ADC_b0-800-2000_ in differentiating PTMCs from benign thyroid nodules.

### Comparison of diagnostic ability between ADC_b0-800-2000_ and US in differentiating nodules of papillary thyroid carcinoma from benign thyroid nodules

According the ROC analysis of high b-value DWI in the study and the previously published US diagnostic criteria of TI-RADS, some important diagnostic evaluation indexes and ROC were compared between ADC_b0-800-2000_ and US in differentiating PTCs and PTMCs from benign thyroid nodules. Generally, ADC_b0-800-2000_ had significantly better diagnostic ability than US in discriminating PTCs (AUC: 0.944 vs. 0.829; Youden's index: 0.82 vs. 0.66; *P* = 0.002) and PTMCs (AUC: 0.940 vs. 0.839; Youden's index: 0.79 vs. 0.68; *P* = 0.005) from benign thyroid nodules (Tables 5 and 6 and Fig 7C and 7D). According to difference degree, the first three diagnostic evaluation indexes highlighting the superiority of ADC_b0-800-2000_ over US were false-positive rate (14.52% vs. 32.26% for PTCs and 14.52% vs. 32.26% for PTMCs), specificity (85.48% vs. 67.74% for PTCs and the same for PTMCs) and positive predict value (PPV) (84.75% vs. 71.83% for PTCs and 77.50% vs. 62.26% for PTMCs).

**Table 5:** Comparison of the diagnostic ability between ADC_b0-800-2000_ (mm^2^/sec) and US in differentiating PTCs from benign thyroid nodules.

Imaging diagnosis *		Pathological results		AUC	Sensitivity (%)	Specificity (%)	PPV (%)	NPV (%)	FPR (%)	FNR (%)	Youden's index
		PTCs (n = 52)	Benign nodules (n = 62)								
ADC_b0-800-2000_, mm^2^/sec	PTCs	50	9	0.944	96.15	85.48	84.75	96.36	14.52	3.85	0.82
	Benign nodules	2	53								
US	PTCs	51	20	0.829	98.10	67.74	71.83	97.67	32.26	1.90	0.66
	Benign nodules	1	42								

* According the ROC analysis, ADC_b0-800-2000_ ≤ 1.16 mm^2^/sec was provided as a cutoff value for diagnosing malignant thyroid nodules (PTCs) and ADC_b0-800-2000_ > 1.16 mm^2^/sec for diagnosing benign nodules. Based on the previously published US criteria of TI-RADS, nodules with ≥ 4a were diagnosed as suspicious malignancies (PTCs) and nodules with 2 or 3 as benign nodules. Abbreviations: ADC, apparent diffusion coefficient; US, ultrasound; PTCs, papillary thyroid carcinomas; PPV, positive predict value; NPV, negative predict value; FPR, False-positive rate; FNR, False-negative rate.

**Table 6:** Comparison of the diagnostic ability between ADCb0-800-2000 (mm^2^/sec) and US in differentiating PTMCs from benign thyroid nodules.

Imaging diagnosis *		Pathological results		AUC	Sensitivity (%)	Specificity (%)	PPV (%)	NPV (%)	FPR (%)	FNR (%)	Youden's index
		PTMCs (n = 33)	Benign nodules (n = 62)								
ADC_b0-800-2000_, mm^2^/sec	PTMCs	31	9	0.940	93.94	85.48	77.50	96.36	14.52	6.06	0.79
	Benign nodules	2	53								
US	PTMCs	33	20	0.839	100	67.74	62.26	100	32.26	0	0.68
	Benign nodules	0	42								

* According the ROC analysis, ADC_b0-800-2000_ ≤ 1.16 mm^2^/sec was provided as a cutoff value for diagnosing malignant thyroid nodules (PTMCs) and ADC_b0-800-2000_ > 1.16 mm^2^/sec for diagnosing benign nodules. Based on the previously published US criteria of TI-RADS, nodules with ≥ 4a were diagnosed as suspicious malignancies (PTMCs) and nodules with 2 or 3 as benign nodules. Abbreviations: ADC, apparent diffusion coefficient; US, ultrasound; PTMCs, papillary thyroid microcarcinomas; PPV, positive predict value; NPV, negative predict value; FPR, False-positive rate; FNR, False-negative rate.

### Reproducibility of SIR and ADC measurement

SIRs and ADCs of the 62 benign thyroid nodules and 52 PTCs (including 33 PTMCs) were reevaluated by two readers (Q. J. Wang and Y. Guo), 6 months after the initial observation. In the benign thyroid nodules, the intraobserver reproducibility was good for SIR_b0_ (ICC, 0.913; 95% CI, 0.856–0.948), SIR_b800_ (ICC,0.728; 95% CI, 0.549–0.836), SIR_b2000_ (ICC, 0.786; 95% CI, 0.644–0.871), ADC_b0-800_ (ICC, 0.893; 95% CI, 0.822–0.936), ADC_b0-2000_ (ICC, 0.892; 95% CI, 0.821–0.935) and ADC_b0-800-2000_ (ICC, 0.892; 95% CI, 0.822–0.935). In the PTCs, the intraobserver reproducibility was excellent for SIR_b0_ (ICC, 0.892; 95% CI, 0.813–0.938), SIR_b800_ (ICC, 0.897; 95% CI, 0.820–0.941), SIR_b2000_ (ICC, 0.907; 95% CI, 0.837–0.946), ADC_b0-800_ (ICC, 0.882; 95% CI, 0.795–0.932), ADC_b0-2000_ (ICC, 0.902; 95% CI, 0.829–0.944) and ADC_b0-800-2000_ (ICC, 0.907; 95% CI, 0.839–0.947).

## Discussion

Our study results revealed that both PTCs and PTMCs show lower SIR_b0_ but higher SIR_b2000_ than benign thyroid nodules. ADC values of both PTCs and PTMCs were lower than that of benign lesions with the same b-value. ADC_b0-800-2000_ had the highest diagnostic ability in discriminating both PTCs and PTMCs from thyroid benign nodules among all these diagnostic indicators. Compared with US, ADC_b0-800-2000_ can obviously reduce false-positive rate and improve the general diagnostic accuracy.

The reason that SIR_b0_ of both PTCs and PTMCs was lower than that of benign thyroid nodules may be due to PTC's pathological features of fibrosis and microcalcification [17], which usually presented as low signal intensity on T2WI. Therefore, PTCs and PTMCs show relatively low SIR on DWI with b-value of 0 sec/mm^2^ because of the similar signal characteristics between T2WI and DWI with b-value of 0 sec/mm^2^. The pathological reflection of SIR_b0_ for PTCs and PTMCs can improve the specificity in differentiating malignant from benign thyroid nodules. Nevertheless, it should be noted that the relatively low sensitivity of SIR_b0_ maybe lead to relatively high missed diagnosis of PTCs and PTMCs.

The mean SIR of benign thyroid nodules decreased with increasing b-values from 0 to 800 and 2000 sec/mm^2^, but the mean SIR of both PTCs and PTMCs increased with the increasing b-values. This obvious SIR difference between benign thyroid nodules and PTCs or PTMCs may help inexperienced radiologists sensitively detect PTCs or PTMCs with the aid of high signal intensities on DWI with b-value of 2000 sec/mm^2^. However, it should be concerned that the specificity of SIR_b2000_ was apparently lower than that of SIR_b0_ for both PTCs and PTMCs, which indicates a high risk of misdiagnosis in differentiating PTCs or PTMCs from benign thyroid nodules only by using SNR_b2000_. We suspect that the relative low specificity of SIR_b2000_ may be due to the reason that SIR_b2000_ cannot present the features of fibrosis and microcalcification in PTCs and PTMCs, at least not as well as SIR_b0_ can. In consideration of no significant difference in diagnostic ability between SIR_b0_ and SIR_b2000_ and their relatively low AUCs, we consider that neither of them is an ideal diagnostic indicator.

Some studies [4–6] and two systematic reviews [7, 8] have confirmed that ADC of malignant thyroid nodules is significantly smaller than that of benign thyroid nodules. However, the b-values used in all these previous studies were not more than 1000 sec/mm^2^. A recent meta-analysis about DWI in differentiating malignant from benign thyroid nodules showed that AUC was 0.95 and the pooled weighted sensitivity and specificity were 90% and 95%, respectively [8]. In our study, the AUC of ADC_b0-800-2000_ mm^2^/sec (0.944) in diagnosing PTCs is similar with the results of the meta-analysis, whereas, the sensitivity (96.15%) is higher than the pooled weighted sensitivity and the specificity (85.48%) is lower than the pooled weighted specificity [8]. This higher sensitivity in our study maybe due to the increased diffusion-weighting and diminished T2 shine-through in ADC maps with higher b-value, which can increase image contrast between PTCs and benign thyroid nodules. The relatively low specificity in our study may be mainly due to a relatively high percentage of the nodules related with hashimoto's thyroiditis in benign thyroid nodules. The previous pathological research have shown that either nodular hashimoto's thyroiditis or other benign nodules complicated by hashimoto's thyroiditis are infiltrated by the large number of lymphocytes, which results in a high nucleus-plasma ratio and high cellularity and then similar to those of PTCs [18]. So, the diagnosis of nodules complicated by hashimoto's thyroiditis should not only depend on DWI with high b-value, but also on other imaging protocols, such as DWI with b-value of 0 sec/mm^2^ or T2WI for identifying the characteristic fibrosis and microcalcification for PTCs and PTMCs. Although our study showed that ADC_b0-800-2000_ had the highest AUC values among all these diagnostic indicators in each subgroup, there was no significantly diagnostic difference between the ADC_b0-800-2000_ and ADC_b0-2000_. So, DWI with high b-value of 2000 sec/mm^2^ can be used alone or can be combined DWI with multiple b-values to analyze thyroid nodules.

As far as we know, our study is the first to exclusively assess the value of DWI in detecting and diagnosing PTMCs. PTMCs have recently become the most common pathological type of thyroid cancer and most PTMCs have benign behavior, little clinical significance, and do not affect patients’ survival [3, 19]. Therefore, the ATA guidelines recommend neither diagnostic US-guided FNAB nor immediate surgical resection for PTMCs [3]. However, aggressive behaviors including extra thyroidal extension, neck lymph node metastasis and distant metastasis can be found in some PTMCs patients [3]. Due to reduction of ultrasonic malignant features, however, conventional US may be not an ideal diagnostic and surveillance method in detecting aggressive behavior of PTMCs [20]. It will be important for PTMCs management if a reliable noninvasive imaging method can be used to identify PTMCs which have a tendency to progress. But before we do that, a diagnostic study is needed to confirm the imaging method can accurately diagnose PTCs and PTMCs, as the ATA guidelines suggest that recommendations for differentiated thyroid cancer should be based on the high-quality evidence from diagnostic accuracy studies [3]. Based on the multi-shot RESOLVE DWI and high in-plane resolution, the high b-value DWI can clearly show almost all the PTMCs larger than 2.0 mm and reach a high level of accuracy with the AUC of 0.940, sensitivity of 93.94% and specificity of 85.48%. The general diagnostic ability of ADC_b0-800-2000_ is significantly better than that of US, especially in the false-positive rate. This illustrates that the high b-value not only can be used as a reliable diagnostic method in PTMCs itself, but also can effectively reduce unnecessary supervision for some micronodules and distinctly relieve psychological burden in some patients in the clinical scenario of thyroid nodules. However, it should be noted that in this study neither MRI nor US can detect the PTMCs less than or equal to 2.0 mm. This indicates that both MRI and US based on respectively current evaluation methods are at a high risk of missing thyroid nodules less than 2.0 mm.

Our study results also revealed that high b-value has a good measurement reproducibility of SIR and ADC. This is very important when high b-value is used as a surveillance method in repeatedly examining PTMCs for detecting any aggressive behaviors. By using RESOVE DWI with conventional b-values (b-values of 0, 400 and 800 sec/mm^2^) and histogram analysis of ADC maps, Schob, S., et al. [13] have found that ADC histogram skewness and kurtosis are able to differentiate nodal negative from nodal positive thyroid carcinoma and can predict prognostically relevant information in thyroid cancer. Compared to common ADC parameters, ADC histogram analysis can give more detailed information on diffusion characteristics of thyroid carcinoma, such as cellularity and proliferative activity. So, if combined with the ADC histogram the high b-value RESOVE DWI has an enormous potential to be used as an active surveillance management approach to PTMCs.

Our work has some limitations. Firstly, this study was a preliminary and single-center study and the patient samples were of insufficient size. A comprehensive and multicenter study involving a larger population is clearly warranted to derive more solid and convincing imaging indicators of PTCs or PTMCs. Secondly, other histological types of thyroid malignant nodules, such as poorly differentiated carcinoma, undifferentiated carcinoma and medullary carcinoma, were not included in our study. Because follicular variant of papillary thyroid carcinoma has been reclassified as noninvasive follicular thyroid neoplasm with papillary-like nuclear features (NIFTP) rather than a conventional thyroid cancer [21], it was also not included in our study. Though PTC accounts for 90% of all thyroid malignant lesions, studies including broader histological types of thyroid malignant lesions will be more representative for application of high b-value DWI in thyroid lesions. Thirdly, only one high b-value (2000 sec/mm^2^) was selected in our study. The reason we chose the b-value of 2000 sec/mm^2^ is because that previous studies have confirmed that b-values in the range of 1500–2500 sec/mm^2^ (but neither lower nor higher) are optimal for prostate cancer detection and diagnosis [14, 15, 22]. Considering that both of the thyroid gland and prostate gland are glandular organs, so we use b-value of 2000 sec/mm^2^ for the high b-value DWI. However, a broader range of b-values for DWI on thyroid imaging should be investigated to determine the optimal b-value of DWI in distinguishing malignant from benign thyroid nodules. Finally, there is a representative issue about the comparison on diagnostic ability between high b-value DWI and US in differentiating nodules of papillary thyroid carcinoma from benign thyroid nodules. Because this comparison was based on the enrolled patients, thyroid lesions with TI-RADS 0 and 1 were not included in the comparison. Furthermore, from the perspective of population inclusion criteria, only these patients planned for US-guided FNAB or thyroid surgery due to thyroid nodules were consecutively enrolled to a thyroid MRI with high b-value DWI. Therefore, the relatively restricted range of case selection maybe limits the reliability of this comparison.

## Conclusions

High b-value (2000 sec/mm^2^) DWI can contribute to differentiating PTCs and PTMCs from benign thyroid nodules with a high level of diagnostic accuracy and has the potential to work as a reliable noninvasive imaging method for PTMCs over an active surveillance.

## Supporting information

S1 Dataset The study's underlying original data. (XLS)
